# Optical Coherence Tomography Parameters as Predictors of Treatment Response to Eplerenone in Central Serous Chorioretinopathy

**DOI:** 10.3390/jcm8091271

**Published:** 2019-08-22

**Authors:** Enrico Borrelli, Biancamaria Zuccaro, Ilaria Zucchiatti, Mariacristina Parravano, Lea Querques, Eliana Costanzo, Riccardo Sacconi, Francesco Prascina, Fabio Scarinci, Francesco Bandello, Giuseppe Querques

**Affiliations:** 1 Department of Ophthalmology, University Vita-Salute, IRCCS Ospedale San Raffaele, Milan 20132, Italy; 2 IRCCS-Fondazione Bietti, Rome 00198, Italy

**Keywords:** medical retina, retinopathy, central serous chorioretinopathy, eplerenone, optical coherence tomography, choroid, retina, retinal imaging, ocular biomarkers

## Abstract

**Purpose**: To present data on clinical response to eplerenone over a 1-year period in patients with central serous chorioretinopathy (CSC), and to evaluate optical coherence tomography (OCT) variables as predictors of treatment response at 3- and 12-month follow-up visits. **Methods**: Patients with acute or chronic CSC treated with eplerenone were retrospectively included. Clinical and imaging characteristics were recorded at baseline and at the 3-month and 12-month follow-up visits. Changes from baseline in quantitative measurements were calculated at each follow-up. Logistic regression analysis was computed to correlate clinical and OCT parameters at baseline with response to treatment at 3 and 12 months of follow-up. **Results**: A total of 50 eyes of 50 patients were included in the study. Mean ± SD best corrected visual acuity (BCVA) was 0.20 ± 0.14 Logarithm of the Minimum Angle of Resolution (LogMAR) at baseline and significantly improved at both the 3-month (0.12 ± 0.13 LogMAR, *p* < 0.0001) and 12-month (0.10 ± 0.12 LogMAR, *p* < 0.0001) follow-up visits. At the 3-month follow-up visit, 25 out of 50 eyes (50.0%) demonstrated macular complete subretinal fluid (SRF) resolution, while 13 eyes (26.0%) showed macular partial SRF resolution, and 12 eyes (24%) had neither partial nor complete macular SRF resolution. Among those patients with macular partial or complete SRF resolution at 3 months and thus not shifted to photodynamic therapy, 36 out of 38 cases had macular complete SRF resolution at the 12-month follow-up visit. There was a significant change from baseline at both follow-up visits in all anatomical OCT parameters (except for reduction in choroidal thickness that did not reach the statistical significance at the 12-month follow-up visit). Several OCT parameters at baseline were independently significant predictors for macular subretinal fluid complete resolution at 3 months, including (i) a thicker subfoveal choroidal thickness; (ii) a smaller subretinal fluid maximum diameter; (iii) a lower number of serous pigment epithelium detachments; and (iv) a lower number of intraretinal hyperreflective foci. **Conclusion**: Treatment with eplerenone in CSC patients is confirmed to be beneficial for both anatomical and functional outcomes. We identified attractive OCT metrics that could potentially be used as a tool to select patients who might mainly benefit from this treatment.

## 1. Introduction

Central serous chorioretinopathy (CSC) is a common cause of central vision loss that typically affects men in their third to fifth decades [1]. This disorder is usually characterized by serous detachments of the neurosensory retina associated with retinal pigment epithelium (RPE) alterations and choroidal vessel dilation. Acute episodes may resolve or progress to the chronic form of this disorder [2], which is characterized by persistent or recurrent detachments of the neuroretina [3].

Although the CSC pathogenesis is multifactorial and not completely understood, mineralocorticoid receptors (MR) have been implicated in the pathological process of this disorder. In detail, MR stimulation was demonstrated to induce CSC-like manifestations in animal models, which can be reversed by MR antagonist (MRA) treatment [4]. Moreover, genetic variants of the mineralocorticoid receptor gene were associated with an increased risk for CSC [5]. Assuming this, two MR antagonists (spironolactone and eplerenone) have been used in the treatment of acute and chronic CSC with promising results in terms of functional and anatomical outcomes [6,7,8,9,10,11,12,13]. Furthermore, while spironolactone may be characterized by some side effects, including reduced libido, gynecomastia, and endocrine abnormalities such as glucose and cortisol elevation, eplerenone has a more favorable side effect profile [14].

Over the past 20 years, optical coherence tomography (OCT) has become an indispensable tool for the diagnosis and follow-up of patients with CSC. Furthermore, imaging analysis granted the identification of predictive factors on the efficacy of MRA in CSC eyes. Our group demonstrated that the presence of hotspot on indocyanine green angiography (ICGA) was associated with a better response to eplerenone [15]. Furthermore, Bousquet and colleagues [16] employed OCT analysis to investigate choroidal thickness and central macular thickness as predictive factors of response to MRA after a relative short follow-up (3 to 6 months after treatment starting). The authors demonstrated that a thicker choroid at baseline was associated with a better response to treatment.

The aim of this report is to present data on clinical response to eplerenone over a 1-year period. Furthermore, we evaluated OCT variables as predictors of treatment response at 3- and 12-month follow-up visits. This might reveal attractive metrics that could potentially be used as a tool to identify patients who may mainly benefit from this treatment and should be included in early intervention trials.

## 2. Methods

### 2.1. Study Participants

This was a multicenter, retrospective cohort study that adhered to the tenets of the Declaration of Helsinki. The study adhered to the 1964 Helsinki declaration and its later amendments. Informed consent was obtained from all individual participants included in the study and it was approved by the Local Institutional Review Board (IRB).

The authors in this study identified patients with acute (first episode with visual symptoms for less than 12 weeks) or chronic CSC as determined by clinical examination and SD OCT, as previously shown [6,7,8,9,10,11,12,13]. Fluorescein and indocyanine green angiographies were performed in select cases to support the diagnosis.

A minimum of three annual OCT scans were required to ensure that all cases had at least a baseline OCT (designated as the first visit in which the patient started the treatment with eplerenone) and an OCT at 3 and 12 months of follow-up. All patients received a complete ophthalmologic examination at each visit, which included the measurement of best corrected visual acuity (BCVA), intraocular pressure (IOP), and dilated ophthalmoscopy.

Exclusion criteria were: (i) a pre-existing retinal disorder that was likely to confound OCT measurements, including diabetic retinopathy, age-related macular degeneration, vitreomacular disorders; (ii) presence of choroidal neovascularization (CNV) or RPE atrophy; and (iii) presence of cystic degeneration on structural OCT. Eyes with prior photodynamic therapy (PDT) were included in the study, as long as they were not actively receiving treatment during the first 3 months of the study period and had not received treatment within 6 months of eplerenone initiation.

All the identified patients were treated with oral eplerenone and per our standard therapeutic approach, the treatment regimen consisted in oral eplerenone 25 mg/day for a week followed by 50 mg/day for 4 weeks. All included patients were revaluated at the end of 5-week therapy with complete ophthalmologic examination. The 50 mg/day eplerenone treatment was thus continued for other 7 weeks depending on the presence of subretinal fluid (SRF) at structural SD-OCT scans. At the 3-month follow-up visit, patients with neither complete or partial SRF resolution were shifted to PDT. We considered a total reabsorption of SRF as a complete response to the treatment, and a reduction of at least 50% of SRF from baseline as a partial response, as previously described [15].

Eplerenone was started after assessment and approval by the patient’s primary care provider. Treatment tolerance was assessed by blood analyses of kaliemia and creatinine performed at baseline and every 4 weeks. The treatment was stopped in case of kaliemia increase (>5 mmol/L) or creatinine clearance rate decrease (<60 mL/min).

Among those patients who had a complete resolution within 1 year, we reviewed the clinical outcomes of patients who relapsed at a later time and were again treated with eplerenone.

### 2.2. Imaging Protocol

Patients underwent spectral-domain (SD)-OCT imaging using the Heidelberg Spectralis device (Heidelberg Engineering, Heidelberg, Germany) with high-resolution (HR), which may obtain OCT scans with an axial resolution of 7 μm in tissue, and a lateral resolution at the retinal surface estimated at approximately 5 μm, as well as an improved visualization of the choroid. Each set of SD-OCT scans consisted of 19 B-scans, each of which comprised 24 averaged scans, covering approximately a 5.5 × 4.5 mm area centered on the fovea. Furthermore, a single horizontal B-scan with HR and enhanced depth imaging (EDI) mode was obtained. To be included in the analysis, a signal strength of at least 25 was required (the manufacturer manual recommended 15 as the borderline quality score) [17].

### 2.3. OCT Analysis

**Figure 1:**
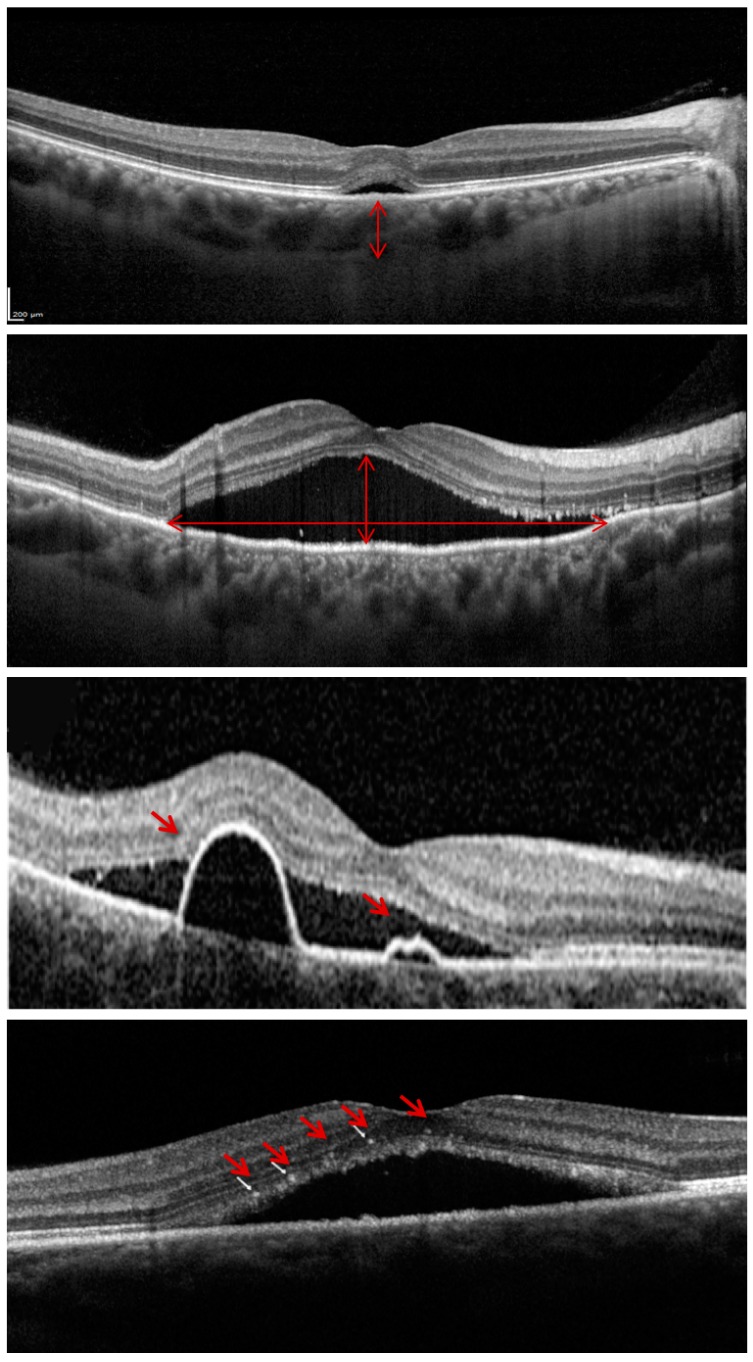
Representation of the optical coherence tomography (OCT) variables assessed at baseline and follow-up visits. In order to provide a comprehensive quantification of the subretinal fluid amount, we measured the foveal subretinal fluid (SRF) height (on the horizontal scan passing through the fovea), the macular maximum SRF height, and the macular maximum SRF diameter (on the top image the two arrows illustrate these measurements). The latter two assessments were performed considering all the OCT B-scans throughout the macula. Furthermore, the number of serous pigment epithelium detachments and intraretinal hyperreflective foci (indicated with the red arrows in the middle and bottom images, respectively) were quantified.

The OCT images were reviewed for qualitative features and analyzed for quantitative measures using the Spectralis built-in analysis software (Heidelberg Eye Explorer Version 6.0, Heidelberg Engineering GmbH, Heidelberg, Germany). The following measurements were performed at baseline and at each follow-up visit, as previously shown [18,19]: (i) subfoveal choroidal thickness; (ii) foveal SRF height; (iii) macular maximum SRF height; (iv) macular maximum SRF diameter; (v) number of pigment epithelium detachments (PEDs); and (vi) number of intraretinal hyperreflective foci (Figure 1). In detail, the (i) and (ii) measurements were performed on the horizontal scan passing through the fovea (the EDI scan for the choroidal thickness), while the other assessments were performed considering all the OCT B-scans throughout the macula. Finally, the central macular thickness was obtained from the instrument built-in software, as previously shown [20].

### 2.4. Statistical Analysis

To detect departures from normality distribution, a Shapiro–Wilk’s test was performed for all variables.

Changes from baseline in quantitative measurements were calculated at each follow-up. To analyze these changes, the related-samples Wilcoxon Signed Rank test was performed. Furthermore, in the comparison between 12-month and baseline values, we excluded those patients undergoing PDT between 3-month and 12-month follow-up visits.

The clinical and OCT parameters at baseline were correlated with response to treatment at 3 and 12 months of follow-up using logistic regression analysis.

Statistical calculations were performed using Statistical Package for Social Sciences (version 20.0, SPSS Inc., Chicago, IL, USA).

*p* Value < 0.05 was considered statistically significant.

## 3. Results

A total of 50 eyes of 50 patients (five females, 45 males) with acute or chronic CSC met the inclusion criteria and were included in the study. Table 1 summarizes clinical characteristics of enrolled patients at baseline.

**Figure 2:**
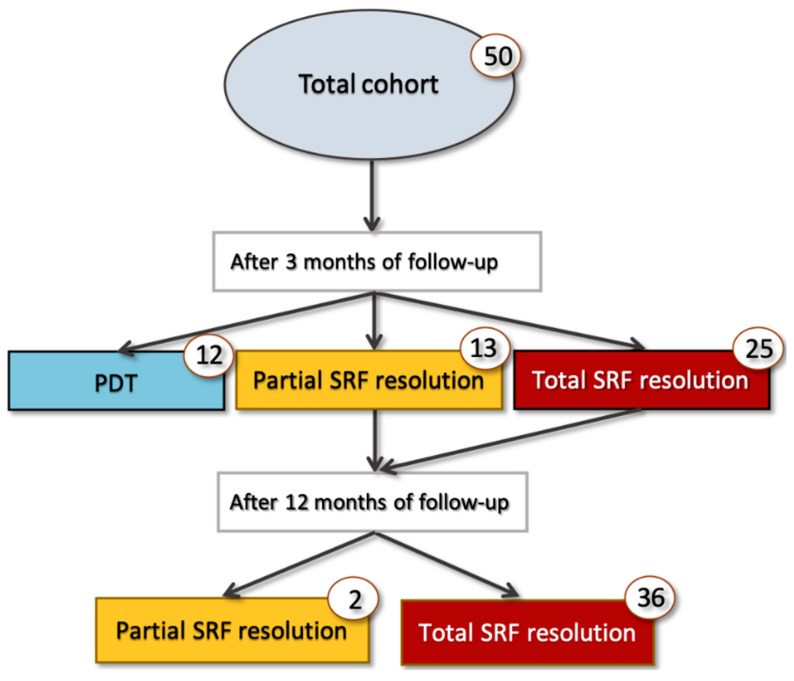
Cohort flowchart of cases’ progression during 1-year follow-up. PDT: Photodynamic therapy.

**Figure 3:**
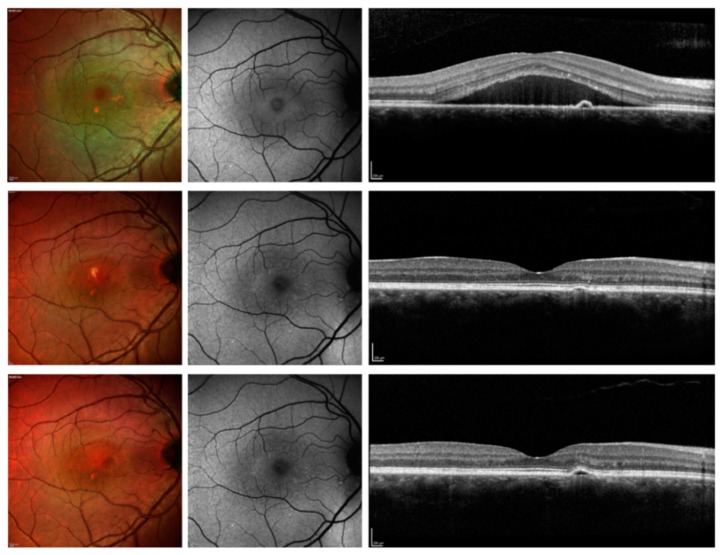
Multimodal imaging of patients with CSC and resolved SRF after 3 months. Top panel: before therapy; middle panel: 3-month follow-up visit; top panel: 12-month follow-up. (**Top panel**) The multicolor image (left image) shows areas of retinal pigment epithelium (RPE) alteration with a region characterized by SRF. The blue fundus autofluorescence image (central image) illustrates a reduced foveal hypoautofluorescence secondary to the presence of SRF. The structural optical coherence tomography B-scan (right image) confirms the presence of SRF. (**Middle panel**) After three months of treatment with eplerenone, the SRF was resolved. The resolution was still present at the 12-month follow-up visit **(Bottom panel**).

**Figure 4:**
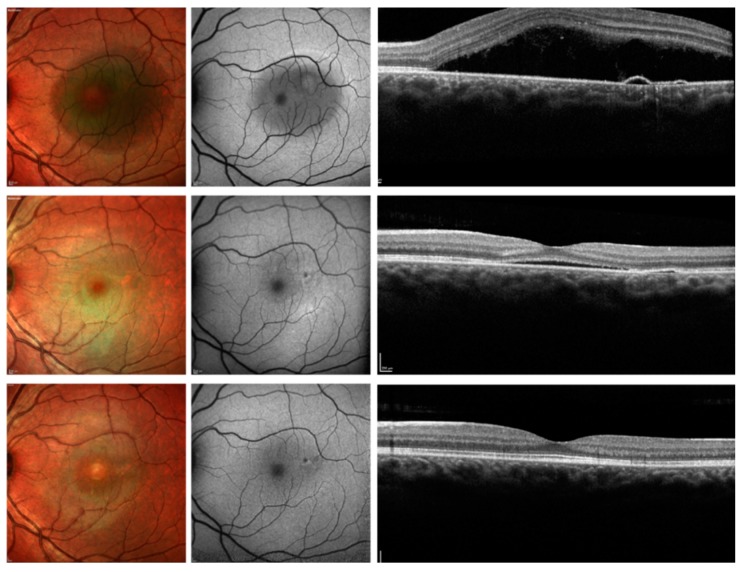
Multimodal imaging of patients with CSC and resolved SRF after 12 months. Top panel: before therapy; middle panel: 3-month follow-up visit; top panel: 12-month follow-up. (**Top panel**) The multicolor image (left image) shows areas of RPE mottling with a region characterized by subretinal fluid (SRF). The blue fundus autofluorescence image (central image) illustrates a reduced macular autofluorescence because of the presence of a large amount of SRF, as confirmed by the structural optical coherence tomography B-scan (right image). (**Middle panel**) After three months of treatment with eplerenone, the SRF was reduced, but still present. The complete resolution was achieved at the 12-month follow-up visit (**Bottom panel**).

At the 3-month follow-up visit, 25 out of 50 eyes (50.0%) demonstrated macular complete SRF resolution, while 13 eyes (26.0%) showed macular partial SRF resolution, and 12 eyes (24%) had neither partial nor complete macular SRF resolution (Figure 2 and Figure 3). Among those patients with macular partial or complete SRF resolution at 3 months and thus not shifted to PDT, 36 out of 38 cases had macular complete SRF resolution at the 12-month follow-up visit (Figure 2 and Figure 4). Considering the foveal complete SRF resolution (analysis performed on the OCT B-scan passing through the fovea), this was reached in 28 and 36 patients, at the 3-month and 12-month follow-up visits, respectively. Treatment with eplerenone was continued for 2.5 ± 0.8 months.

Considering distinctly acute and chronic CSC, eight out of 14 acute CSC (%) and 17 out of 36 chronic CSC (%) demonstrated macular complete SRF resolution at 3 months. At 12 months and considering only patients not shifted to PDT, 12 out of 12 acute CSC and 24 out of 26 chronic cases had a macular complete SRF resolution.

### 3.1. Clinical Changes During Follow-up

Mean ± SD BCVA was 0.20 ± 0.14 LogMAR at baseline and significantly improved at both 3-month (0.12 ± 0.13 LogMAR, *p* < 0.0001) and 12-month (0.10 ± 0.12 LogMAR, *p* < 0.0001) follow-up visits. Baseline mean central macular thickness was 392.3 ± 129.5 µm and significantly decreased at both the 3-month (298.3 ± 86.7 µm, *p* < 0.0001) and 12-month (259.9 ± 62.5 µm, *p* < 0.0001) follow-up visits. There was a significant change from baseline at first follow-up in all anatomical OCT parameters (Table 2). While changes were still statistically significant at 12 months for the other parameters, reduction in choroidal thickness (*p* = 0.124) was still notable, but not statistically significant at the 12-month follow-up visit.

### 3.2. Logistic Regression Analysis

Logistic regression analysis, assessing the importance of OCT variables and clinical characteristics, revealed that in this dataset, only a thicker subfoveal choroidal thickness was a univariate significant predictor for foveal SRF complete resolution at 3 months (Table 3). On the contrary, different OCT parameters at baseline were an independently significant predictor for macular SRF complete resolution at 3 months, including (i) a thicker subfoveal choroidal thickness; (ii) a narrower subretinal fluid maximum diameter; (iii) a lower number of serous PEDs; and (iv) a lower number of intraretinal hyperreflective foci (HF) (Table 4). At 1 year of follow-up, neither OCT variables nor clinical characteristics were associated with macular and foveal SRF resolution at 1 year.

### 3.3. Subanalysis in Relapsing Patients

**Figure 5:**
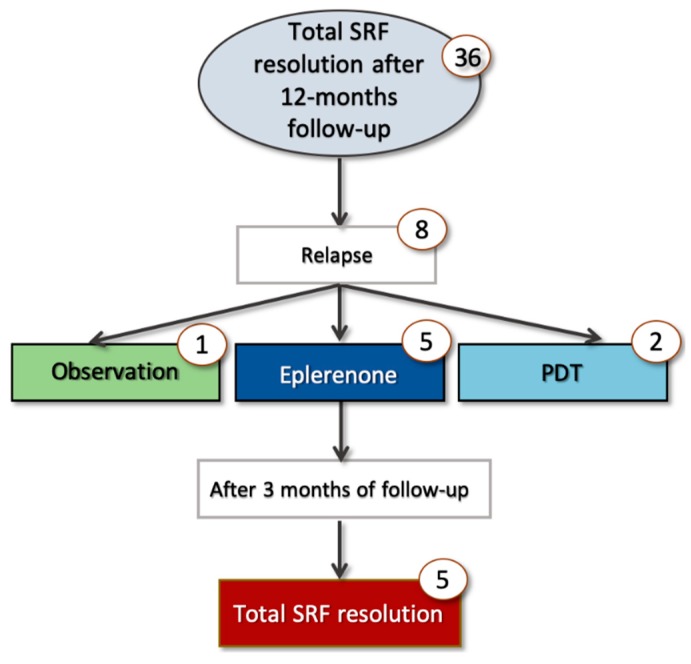
Cohort flowchart of cases relapsing at a later time.

In a subanalysis considering only those patients obtaining macular complete SRF resolution within 1 year, eight out of 36 patients (22.2 %) presented a recurrence at a later time (Figure 5). Recurrences occurred 8.5 ± 6.5 months after the 12-month follow-up visit (two patients within 1 year after discontinuing treatment). Of these patients, five were retreated with eplerenone, while two patients underwent PDT and in one patient, watch-and-wait approach was used. In all cases retreated with eplerenone, a complete SRF resolution was observed within 3 months.

## 4. Discussion

In this retrospective, longitudinal study, we described functional and anatomical response to eplerenone over a 1-year period. Overall, we observed that this treatment is effective in acute/chronic CSC eyes. In addition, this treatment was revealed to be useful also in those eyes relapsing at a later time and again undergoing treatment with eplerenone. More importantly, our analysis granted the identification of different OCT variables as predictors of treatment response, which resulted independent of other clinical characteristics, such as previous treatments and disease duration.

Several previous notable studies have reported on the outcomes of MRA treatment in CSC patients [6,7,8,9,10,11,12]. Ghadiali et al. [11] retrospectively investigated 11 patients (12 eyes) with chronic CSC treated with MRA (spironolactone or eplerenone) for 6 to 12 months. The authors demonstrated a significant reduction in SRF at 12 months. Zola and colleagues [12] assessed the clinical outcomes of 16 patients (16 eyes) with chronic CSC undergoing MRA treatment. In the latter study cohort, the mean time to complete foveal SRF resolution was 10.5 ± 8.0 months after treatment initiation and, at 24 months, foveal SRF resolution was achieved in 13 eyes (81%). Toto and colleagues [21] also demonstrated that eplerenone is effective in improving visual function and morphological parameters in eyes with chronic CSC. In their study cohort, a percentage of 71.4% of eyes had resolution of SRF within 2 months, while the remaining 28.6% had resolution at the 2-month follow-up visit. Importantly, the MRA treatment was demonstrated to be effective also in eyes with acute CSC. In a case-control study, our group compared eplerenone therapy with observation alone in patients with acute CSC [8]; we demonstrated that patients affected by acute CSC and treated with eplerenone achieved a greater and faster resolution of SRF compared to the observation group.

Our current results similarly showed that therapy with eplerenone may be effective in CSC patients. We demonstrated that this treatment results in a significant improvement in visual acuity and SRF measures. Furthermore, we displayed that several OCT variables modify throughout the 1-year follow-up. In detail, therapy with eplerenone was associated with a decrease in subfoveal choroidal thickness, as well as a reduction in serous PEDs’ number. Assuming that these variables are known to be expression of the choroidal flow dysregulation [22,23], our results further support the hypothesis of MR involvement in CSC pathogenesis. Furthermore, at the 3-month follow-up visit, a complete resolution of foveal SRF was observed in 56.0% of patients, while a complete resolution of macular SRF was achieved in 50.0% of cases, which is in line with previous works [8,12,16]. Of note, the rate of complete SRF resolution (both foveal and macular) at the 12-month follow-up visit was 94.7%, considering only those patients with partial/complete resolution at 3 months and thus not shifted to PDT. These results suggest that, while not all patients may respond to eplerenone at 3 months, those patients with a partial resolution may still experience an improvement in clinical outcomes in the following months. Notably, this improvement seems to be independent of treatment discontinuance, taking into consideration that eplerenone was stopped after 5 to 12 weeks in our study cohort. This seems to be consistent with the hypothesis that a benefit on anatomical outcomes may persist after treatment suspension, which may be consequent to acquired changes in choroidal flow that persist after treatment cessation. The latter hypothesis is also consistent with the evidence that recurrences are less frequent and appear later in patients previously treated with MRA [24]. Alternatively, this improvement occurring after treatment cessation might be totally independent of eplerenone and might reflect the natural history of the disease.

We add to the literature by reporting OCT variables as predictors of treatment response at 3- and 12-month follow-up visits, even after accounting for confounding factors, such as disease duration and previous treatments. We demonstrated that a thicker subfoveal choroidal thickness at baseline was associated with a better response to treatment at 3 months, which is consistent with previous reports [16,25]. The latter aspect was speculated to be related to a better effect of the drug on choroidal vessels in eyes with thicker choroid [16]. More importantly, we showed that a higher number of serous PEDs and a wider SRF diameter were associated with a poorer response to eplerenone. We propose that these findings are related to an advanced retinal pigment epitheliopathy at baseline in these eyes, with a consequent lower capability of the RPE cells to reabsorb SRF under eplerenone treatment. Similarly, we observed that a greater number of intraretinal hyperreflective foci was correlated with poor treatment response. Although their precise nature is unclear, it has been suggested that HF are the extravasation of lipoproteins or activated microglia with a phagocytized photoreceptor or intraretinally migrated RPE [18]. Accordingly, the presence of these hyperreflective foci may reflect greater damage of the RPE and this may eventually account for our findings.

Although we did demonstrate that several OCT parameters may be used as a tool to identify patients who might mainly benefit from treatment with eplerenone, these parameters were shown to be useful to predict response at 3 months, while we failed to find statistically significant associations between OCT parameters and the response at 12 months. The latter finding may be related to a potential selection bias at the 12-month follow-up visit, assuming that those patients not reaching a partial/complete resolution at 3 months were shifted to PDT. Future prospective larger studies may shed further light on this aspect in order to comprehend whether other OCT parameters may predict treatment response at a later follow-up.

While the focus of the present paper was to characterize the response to eplerenone over a 1-year period, among those patients who had a complete resolution within this interval, we reviewed the clinical outcomes of patients who relapsed at a later time and were again treated with eplerenone. Interestingly, all those patients who relapsed and were treated again with eplerenone experienced a complete SRF resolution within 3 months. A longer prospective longitudinal evaluation on CSC patients may further clarify whether a first complete response to eplerenone is a good indicator of treatment response in successive relapses.

Our study has limitations including its retrospective nature and the relatively small sample size, inevitably resulting in a lack of uniformity. Moreover, patients previously treated with other therapies were included in this retrospective analysis, which further reduces the cohort homogeneity. However, our statistical analysis was aimed at accounting for potential confounding factors, including previous treatments. In addition, excluding patients with previous treatments might have resulted in a potential selection bias. A final limitation is that we did not specifically investigate the repeatability of our measurements. However, the repeatability for these measurements was reported previously and was shown to be excellent [26,27,28].

## 5. Conclusions

In conclusion, this study investigated the clinical outcomes of patients with CSC and treated with eplerenone. We demonstrated that this therapy is effective in treating these patients and that several OCT variables modify throughout the follow-up visits. Importantly, this study highlights the presence of OCT-related parameters as predictors of treatment response to eplerenone. If replicated in future studies, these OCT variables might potentially be used as a tool to identify CSC patients who might mainly benefit from eplerenone and thus should be included in upcoming clinical trials.

## Figures and Tables

**Table 1:** Clinical characteristics of enrolled patients at baseline.

	CSC Patients
Number of eyes enrolled (patients)	50 (50)
Age, years	44.0 ± 8.9
Gender	
M, *n* (%)	45 (90%)
F, *n* (%)	5 (10%)
Race	
M, *n* (%)	50 (100%)
Form	
Acute	14 (28%)
Chronic	36 (72%)
Disease Duration, months	37.8 ± 53.2
Previous Treatments	
PDT	6 (12.2%)
Laser	0 (0%)
Anti-VEGF	0 (0%)
BCVA, LogMAR	0.20 ± 0.14

CSC: Central serous chorioretinopathy. PDT: photodynamic therapy. VEGF: vascular endothelial growth factor. BCVA: best corrected visual acuity. LogMAR: Logarithm of the Minimum Angle of Resolution.

**Table 2:** Tested optical coherence tomography variables throughout the follow-up among 39 patients who were shifted to other treatments during the follow-up.

	Baseline	3 Months	*p* Value	12 Months	*p* Value
Subfoveal Choroidal Thickness, μm	398 (342–468)	370 (329–442)	0.046	396 (309–462)	0.124
Subfoveal Subretinal Fluid Height, μm	169 (52–148)	0 (0–84)	<0.0001	0 (0–24)	<0.0001
Subretinal Fluid maximum Height, μm	182 (97–272)	68 (0–121)	<0.0001	0 (0–58)	<0.0001
Subretinal Fluid Maximum Diameter, μm	2449 (1351–3112)	1065 (0–2307)	<0.0001	0 (0–726)	<0.0001
Pigment Epithelium Detachments, *n*	1 (0–2)	0 (0–1)	0.004	0 (0–1)	0.003
Hyperreflective Foci, *n*	9 (4–18)	5 (3–8)	<0.0001	0 (2–4)	<0.0001

Comparison were computed using the Related-Samples Wilcoxon Signed Rank test. Data are reported as median (interquartile range).

**Table 3:** Logistic regression analysis for potential predictors of foveal complete resolution after treatment.

Predictor for Foveal Complete Resolution	3 Months			12 Months		
	Odds Ratio	95% CI	*p* Value	Odds Ratio	95% CI	*p* Value
Subfoveal Choroidal Thickness	1.012	1.001–1.024	0.030	0.998	0.988–1.008	0.702
Subfoveal Subretinal Fluid Height	0.991	0.974–1.008	0.308	0.988	0.967–1.009	0.266
Subretinal Fluid Maximum Height	1.010	0.987–1.033	0.407	1.019	0.991–1.047	0.180
Subretinal Fluid Maximum Diameter	0.999	0.998–1.000	0.162	1.000	0.999–1.001	0.538
Pigment Epithelium Detachments’ Number	0.693	0.401–1.198	0.189	0.886	0.464–1.691	0.714
Hyperreflective Foci Number	0.972	0.874–1.060	0.543	1.014	0.909–1.110	0.779
Age	1.064	0.989–1.144	0.094	1.012	0.933–1.097	0.780
Disease Duration	0.995	0.982–1.008	0.456	1.004	0.989–1.019	0.618
Previous Treatments	0.971	0.911–1.031	0.515	1.007	0.982–1.032	0.218

**CI:** Confidence interval.

**Table 4:** Logistic regression analysis for potential predictors of complete macular resolution after treatment.

Predictor for Macular Complete Resolution	3 Months (*n* = 50)			12 Months (*n* = 39)		
	Odds Ratio	95% CI	*p* Value	Odds Ratio	95% CI	*p* Value
Subfoveal Choroidal Thickness	1.021	1.004–1.038	0.013	0.991	0.978–1.004	0.158
Subfoveal Subretinal Fluid Height	1.000	0.982–1.018	0.969	0.997	0.978–1.015	0.719
Subretinal Fluid Maximum Height	1.017	0.914–1.017	0.211	1.023	0.994–1.053	0.129
Subretinal Fluid Maximum Diameter	0.996	0.994–0.999	0.013	1.000	0.999–1.001	0.984
Pigment Epithelium Detachments’ Number	0.259	0.088–0.759	0.012	0.817	0.326–2.047	0.667
Hyperreflective Foci Number	0.694	0.349–0.967	0.025	0.938	0.902–1.059	0.331
Age	1.173	0.972–1.232	0.012	1.006	0.923–1.097	0.887
Disease Duration	0.997	0.974–1.020	0.774	1.008	0.993–1.025	0.300
Previous Treatments	0.871	0.823–0.919	0.614	1.005	0.979–1.028	0.225

**CI:** Confidence interval.

## References

[1] Kitzmann A.S., Pulido J.S., Diehl N.N., Hodge D.O., Burke J.P. (2008). The Incidence of Central Serous Chorioretinopathy in Olmsted County, Minnesota, 1980–2002. Ophthalmology.

[2] Quin G., Liew G., Ho I.-V., Gillies M., Fraser-Bell S. (2013). Diagnosis and interventions for central serous chorioretinopathy: Review and update. Clin. Exp. Ophthalmol..

[3] Loo R.H., Scott I.U., Flynn H.W., Gass J.D.M., Murray T.G., Lewis M.L., Rosenfeld P.J., Smiddy W.E. (2002). Factors associated with reduced visual acuity during long-term follow-up of patients with idiopathic central serous chorioretinopathy. Retina.

[4] Zhao M., Célérier I., Bousquet E., Jeanny J.-C., Jonet L., Savoldelli M., Offret O., Curan A., Farman N., Jaisser F. (2012). Mineralocorticoid receptor is involved in rat and human ocular chorioretinopathy. J. Clin. Investig..

[5] Van Dijk E.H.C., Schellevis R.L., Van Bergen M.G.J.M., Breukink M.B., Altay L., Scholz P., Fauser S., Meijer O.C., Hoyng C.B., Hollander A.I.D. (2017). Association of a Haplotype in the NR_3_C_2_ Gene, Encoding the Mineralocorticoid Receptor, with Chronic Central Serous Chorioretinopathy. JAMA Ophthalmol..

[6] Schwartz R., Habot-Wilner Z., Martinez M.R., Nutman A., Goldenberg D., Cohen S., Shulman S., Guzner-Gur H., Loewenstein A., Goldstein M. (2017). Eplerenone for chronic central serous chorioretinopathy—A randomized controlled prospective study. Acta Ophthalmol..

[7] Bousquet E., Beydoun T., Rothschild P.R., Bergin C., Zhao M., Batista R., Brandely M.L., Couraud B., Farman N., Gaudric A. (2015). Spironolactone for nonresolving central serous chorioretinopathy a randomized controlled crossover study. Retina.

[8] Zucchiatti I., Sacconi R., Parravano M.C., Costanzo E., Querques L., Montorio D., Bandello F., Querques G. (2018). Eplerenone Versus Observation in the Treatment of Acute Central Serous Chorioretinopathy: A Retrospective Controlled Study. Ophthalmol. Ther..

[9] Breukink M.B., Hollander A.I.D., Keunen J.E., Boon C.J., Hoyng C.B. (2014). The use of eplerenone in therapy-resistant chronic central serous chorioretinopathy. Acta Ophthalmol..

[10] Montorio D., Carnevali A., Sacconi R., Capuano V., Giuffrè C., Rabiolo A., De Vitis L.A., Querques L., Bandello F., Querques G. (2017). Mineralocorticoid receptor antagonists in the treatment of central serous chorioretinopathy. Expert Rev. Ophthalmol..

[11] Ghadiali Q., Jung J.J., Yu S., Patel S.N., Yannuzzi L.A. (2015). Central Serous Chorioretinopathy Treated With Mineralocorticoid Antagonists: A One-Year Pilot Study. Retina.

[12] Zola M., Daruich A., Matet A., Mantel I., Behar-Cohen F. (2018). Two-year follow-up of mineralocorticoid receptor antagonists for chronic central serous chorioretinopathy. Br. J. Ophthalmol..

[13] Van Rijssen T.J., Van Dijk E.H., Yzer S., Ohno-Matsui K., Keunen J.E., Schlingemann R.O., Sivaprasad S., Querques G., Downes S.M., Fauser S. (2019). Central serous chorioretinopathy: Towards an evidence-based treatment guideline. Prog. Retin. Eye Res..

[14] Lainscak M., Pelliccia F., Rosano G., Vitale C., Schiariti M.S.M., Greco C., Speziale G., Gaudio C. (2015). Safety profile of mineralocorticoid receptor antagonists: Spironolactone and eplerenone. Int. J. Cardiol..

[15] Sacconi R., Baldin G., Carnevali A., Querques L., Rabiolo A., Marchini G., Bandello F., Querques G. (2018). Response of central serous chorioretinopathy evaluated by multimodal retinal imaging. Eye.

[16] Bousquet E., Dhundass M., Lejoyeux R., Shinojima A., Krivosic V., Mrejen S., Gaudric A., Tadayoni R. (2019). Predictive Factors of Response to Mineralocorticoid Receptor Antagonists in Nonresolving Central Serous Chorioretinopathy. Am. J. Ophthalmol..

[17] Huang Y., Gangaputra S., Lee K.E., Narkar A.R., Klein R., Klein B.E.K., Meuer S.M., Danis R.P. (2012). Signal Quality Assessment of Retinal Optical Coherence Tomography Images. Investig. Opthalmol. Vis. Sci..

[18] Lee H., Lee J., Chung H., Kim H.C. (2016). Baseline spectral domain optical coherence tomographic hyperreflective foci as a predictor of visual outcome and recurrence for central serous chorioretinopathy. Retina.

[19] Petkovsek D.S., Cherfan D.G., Conti F.F., Hom G.L., Ehlers J.P., Babiuch A.S., Rachitskaya A.V., Kaiser P.K., Schachat A.P., Srivastava S.K. (2019). Eplerenone for the treatment of chronic central serous chorioretinopathy: 3-year clinical experience. Br. J. Ophthalmol..

[20] Comyn O., Heng L.Z., Ikeji F., Bibi K., Hykin P.G., Bainbridge J.W., Patel P.J. (2012). Repeatability of Spectralis OCT Measurements of Macular Thickness and Volume in Diabetic Macular Edema. Investig. Opthalmol. Vis. Sci..

[21] Toto L., D’Aloisio R., Mastropasqua R., Di Antonio L., Di Nicola M., Di Martino G., Evangelista F., Erroi E., Doronzo E., Mariotti C. (2019). Anatomical and Functional Changes of the Retina and the Choroid after Resolved Chronic CSCR. J. Clin. Med..

[22] Hirami Y., Tsujikawa A., Sasahara M., Gotoh N., Tamura H., Otani A., Mandai M., Yoshimura N. (2007). Alterations of retinal pigment epithelium in central serous chorioretinopathy. Clin. Exp. Ophthalmol..

[23] Van Rijssen T.J., Van Dijk E.H.C., Scholz P., Breukink M.B., Blanco-Garavito R., Souied E.H., MacLaren R.E., Querques G., Fauser S., Hoyng C.B. (2019). Patient characteristics of untreated chronic central serous chorioretinopathy patients with focal versus diffuse leakage. Graefe’s Arch. Clin. Exp. Ophthalmol..

[24] Herold T.R., Rist K., Priglinger S.G., Ulbig M.W., Wolf A. (2017). Long-term results and recurrence rates after spironolactone treatment in non-resolving central serous chorio-retinopathy (CSCR). Graefes Arch. Clin. Exp. Ophthalmol..

[25] Gergely R., Kovács I., Schneider M., Resch M., Papp A., Récsán Z., Nagy Z.Z., Ecsedy M. (2017). Mineralocorticoid receptor antagonist treatment in bilateral chronic central serous chorioretinopathy. Retina.

[26] Nassisi M., Fan W., Shi Y., Lei J., Borrelli E., Ip M., Sadda S.R. (2018). Quantity of Intraretinal Hyperreflective Foci in Patients With Intermediate Age-Related Macular Degeneration Correlates with 1-Year Progression. Investig. Opthalmol. Vis. Sci..

[27] Benson S.E., Schlottmann P.G., Bunce C., Xing W., Charteris D.G. (2006). Assessment of the reproducibility and repeatability of a method of grading macular subretinal fluid using optical coherence tomography. Eye.

[28] Ikuno Y., Maruko I., Yasuno Y., Miura M., Sekiryu T., Nishida K., Iida T. (2011). Reproducibility of Retinal and Choroidal Thickness Measurements in Enhanced Depth Imaging and High-Penetration Optical Coherence Tomography. Investig. Opthalmol. Vis. Sci..

